# Re-Examination of the Holotype of *Ganoderma sichuanense* (*Ganodermataceae*, *Polyporales*) and a Clarification of the Identity of Chinese Cultivated Lingzhi

**DOI:** 10.3390/jof9030323

**Published:** 2023-03-05

**Authors:** Zhuo Du, Yi Li, Xin-Cun Wang, Ke Wang, Yi-Jian Yao

**Affiliations:** 1 Fungarium (HMAS), Institute of Microbiology, Chinese Academy of Sciences, Beijing 100101, China; 2 College of Food Science and Engineering, Yangzhou University, Yangzhou 225127, China; 3 State Key Laboratory of Mycology, Institute of Microbiology, Chinese Academy of Sciences, Beijing 100101, China

**Keywords:** DNA sequence, *Ganoderma sichuanense*, *Ganoderma lingzhi*, Lingzhi, nomenclature, type material

## Abstract

The widely cultivated Chinese Lingzhi is a famous fungus with significant medicinal and economic value, which has commonly been misidentified as *Ganoderma lucidum* for a long period of time. The scientific binomial of the fungus is always a hotly debated question that revolves around *G*. *lingzhi* and *G*. *sichuanense*. To interpret the species concept of the taxon, six specific primers for *G*. *sichuanense* and one universal primer were designed. Through directed and nested PCRs, we obtained nine ITS sequences from the holotype (HMAS 42798) of *G*. *sichuanense*. By genome sequencing, the ITS sequence of the first cultivated Lingzhi (HMAS 25103) was assembled. Based on a phylogenetic study of the genus *Ganoderma*, the correct name for widely cultivated *Ganoderma* species in China was confirmed as *G*. *sichuanense*, and *G*. *lingzhi* should be a later synonym.

## 1. Introduction

*Ganoderma* P. Karst. is a cosmopolitan genus established by Karsten [1], based on the generic type *G*. *lucidum* (Curtis) P. Karst from England [2]. The use of *Ganoderma* mushroom in China can be traced back to 6800 years ago [3], and species of *Ganoderma* have had a considerable impact on Chinese history [4]. According to Tai [5], Patouillard first identified *G*. *lucidum* in China in 1907 by the specimens collected from Guizhou Province.

However, based on the morphological characters, such as the thickness of context and diameter of the stipe, Pegler and Yao suggested that the widely cultivated “*G. lucidum*” (as “Lingzhi” or “Ruizhi” in Chinese) in China was not conspecific with the species described in Europe [6]. The result was also supported by phylogenetic evidence [7,8,9,10,11]. In 1959, the first successful cultivation of the fruit bodies of Lingzhi was developed by the Institute of Microbiology, Chinese Academy of Sciences. Approximately 10 years later, under the vigorous promotion of the *Ganoderma* research group at the Institute of Microbiology [12], the cultivation of Lingzhi became an important industry in China and other adjacent countries. At present, this fungus is famous as a traditional Chinese medicine possessing great economic value [13,14,15,16,17].

The scientific binomial for this economically and medicinally important fungus, Lingzhi, has long been controversial, which was considered to be *G*. *lucidum* for a long period in China. Using molecular phylogeny, Wang et al. highlighted that the Asian *G*. *lucidum* specimens were separated from the European *G*. *lucidum* by two individual clades, and the tropical collections from Asian areas represented *G*. *multipileum* D. Hou 1950, while the classification status of the other collections obtained from mainland China, Japan, and Korea was uncertain [11]. Wang et al. recognized the uncertain clade as *G*. *sichuanense* J.D. Zhao & X.Q. Zhang [18,19], which is the *Ganoderma* species widely cultivated in China. However, Cao et al. proposed it as a new species *G*. *lingzhi* Sheng H. Wu, Y. Cao & Y.C. Dai based on a single available internal transcribed spacer (ITS) sequence from the holotype (HMAS 42798) of *G*. *sichuanense* [20]. Yao et al. designated an epitype (HMAS 252081) to interpret the species concept of Lingzhi and secured the position of the holotype of *G*. *sichuanense* (HMAS 42798), both morphologically and molecularly [21]. However, based on the holotype sequence from Cao et al. [20], the epitype was not accepted by some researches [22,23,24]. Yao et al. re-clarified the typification of *G*. *sichuanense* and demonstrated that the epitype of *G*. *sichuanense* was appropriately designated to support the holotype of the name [25]. 

To clarify the confusion of this important fungus, we designed six specific primers for *G*. *sichuanense* and one universal primer to obtain the ITS sequence from the *G*. *sichuanense* holotype (HMAS 42798) (by directed PCR and nested PCR), which is the key point of the hot topic. For the fruit bodies of the first cultivated Lingzhi (HMAS 25103), its DNA had been largely degraded, so genome sequencing was chosen. Based on all these representative sequences, we performed a phylogenetic study of the genus *Ganoderma*, including the type materials of *G*. *sichuanense* (holotype, epitype, and topotype) and *G*. *lingzhi* (holotype). As a result, we can confirm that *G*. *lucidum* is a name mistakenly applied to the widely cultivated *Ganoderma* species in China, that the scientific binomial for Lingzhi is *G*. *sichuanense*, and that the designation of the epitype is necessary to support the holotype because of its poor DNA status. *G*. *lingzhi* is the later synonym of *G*. *sichuanense.*

## 2. Materials and Methods

### 2.1. Specimens

The fungal collections are deposited in the Fungarium of the Institute of Microbiology (HMAS) of the Chinese Academy of Sciences; including the holotype of *G*. *sichuanense* (HMAS 42798), the topotype (HMAS 244431) collected from Panzhihua City (previously “Dukou Shi”) in Sichuan Province, and the first cultivated Lingzhi (HMAS 25103), performed by Zhuang Deng, the daughter and assistant of Professor Shu-Chün Teng, in 1959.

### 2.2. DNA Samples

A total of 24 genomic DNA samples were extracted from the holotype (HMAS 42798) by Xin-Cun Wang and Li Yi in 2010 using various methods, including the CTAB method described in Jiang and Yao [26], the Wizard^®^ Genomic DNA Purification Kit (Promega, U.S.A.), and Chelex 100 Resin (Solarbio, China). The DNA samples were kept in ultra-low temperature freezer (below −80 °C) until use. The additional sampling of the topotype (HMAS 244431) was performed by Zhuo Du separately to avoid any possible contamination, using a DNA Extracting Kit (Cat#: NEP023-1) distributed by Beijing Dingguochangsheng Biotechnology Co. Ltd. (Beijing, China), following the instructions of the manufacturer.

### 2.3. Specific Primer Design, Amplification, and Sequencing

The specific primers for *G*. *sichuanense* were designed based on the sequence alignment of ITS sequences obtained from *G*. *lucidum*, *G*. *multipileum*, *G*. *resinaceum*, *G*. *sichuanense*, *G*. *tropicum,* and *G*. *weberianum*. Primer 3 v. 0.4.0 (http://bioinfo.ut.ee/primer3-0.4.0/, accessed on 15 June 2018) software was employed in combination with manual adjustments. The primers were selected and tested using Primer 5 v. 5.00 and then utilized to perform amplifications (Table 1). The suggested annealing temperature of Primer 5 v. 5.00 was tested in PCR and compared to the conventional temperature of 55 °C, and the latter was adopted throughout the experiment. 

**Figure 1:**
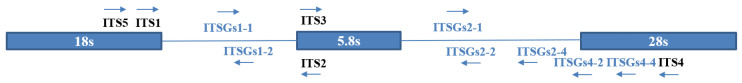
The locations of primers in the internal transcribed spacer (ITS) region; the arrowheads represent the 3’end of each primer (primers designed in this study are in blue).

The primers used in the amplifications included ITS5, ITS1, and ITS4 for both ends, ITS2 and ITS3 were utilized for the internal positions of the whole length of ITS1-5.8S-ITS2 [27]. ITSGs1-1, ITSGs1-2, ITSGs2-1, ITSGs2-2, ITSGs2-4, and ITSGs4-2, are specific to *G*. *sichuanense* and ITSGs4-4 is a universal primer, all of which were designed in the present study. The sequences and locations of the newly designed primers are presented in Table 1 and Figure 1.

Both directed and nested PCRs with various combinations of primer pairs were used to obtain better results. PCR thermal cycling was performed in 25 μL reaction mixtures containing 1 μL of DNA template, 12.5 μL of 2 × PCR Master Mix, 1 μL of each PCR primer (10 μM), and 9.5 μL of double-distilled H_2_O. For the nested PCR, the second round of reactions consisted of a template at a 1:10 dilution of the first round PCR product. The PCR protocol comprised denaturation at 95 °C for 4 min, followed by 30 cycles of 95 °C for 30 s, 55 °C for 30 s, 72 °C for 1 min, and a final cycle at 72 °C for 10 min.

1. Directed PCR: the following 4 primer combinations were used: ITS1/ITSGs1-2, ITSGs1-1/ITS2, ITS3/ITSGs2-2, and ITSGs2-1/ITS4.
2. Nested PCR: ITS5/ITS4 was followed by 6 different primer pairs as internal primers (Table 2).

Based on the aim of obtaining a full-length ITS sequence, all the PCR products of directed PCRs and two groups of nested PCRs (internal primer sets: ITS1/ITSGs4-2 and ITSGs1-1/ITSGs4-4) were selected to create a sequence. DNA sequencing was performed using an ABI PRISM^®^ 3730XL DNA Analyzer with a BigDye^®^ Terminator Kit v3.1 at the Tsingke Biological Technology Company (Beijing, China) 

### 2.4. Genome Sequencing

The first artificially cultivated Lingzhi (HMAS 25103) was performed in 1959. Due to the long time preservation, the surface was contaminated with other fungi. The DNA condition of the specimen was very poor and heavily disintegrated. All the methods mentioned above used to amplify the DNA fragments from this specimen were unsuccessful; therefore, genome sequencing was performed to obtain the ITS region sequence.

Approximately 50 mg of ground tissue from HMAS 25103 was sent to the Shanghai Biozeron Biotechnology Company (Shanghai, China). DNA extraction was performed by the company using E.Z.N.A.^®^ Stool DNA Kits (OMEGA Bio-tek, Norcross, GA, USA), and the quality was tested on 1% agarose by Covaris M220. Paird-end (PE) libraries were developed using the TruSeq™ DNA Sample Prep Kit. Then a cBot Truseq PE Cluster Kit v3-cBot-HS was utilized to accomplish bridge PCR. Additionally, Illumina sequencing was carried out by a Truseq SBS Kit (300 cycles).

### 2.5. Phylogenetic Analyses

A total of 185 DNA sequences generated by each forward and reverse primer were used to obtain consensus sequences using Seqman v.7.1.0 in the DNASTAR laser gene core suite software (DNASTAR, Madison, WI, USA). The single gene ITS sequences were initially aligned with Clustal W and implemented in MEGA 6 and improved by MAFFT v.7 [28,29]. *Tomophagus colossus* (Fr.) Murrill was selected as the outgroup taxon for all analyses [18,21]. The aligned matrices used for the phylogenetic analyses were maintained in TreeBASE (www.treebase.org; accession number: 30118). 

RAxML-HPC BlackBox v.8.2.10 was performed to construct a maximum likelihood (ML) tree, employing a GTRGAMMA substitution model with 1000 bootstrap replicates [30]. The branch support of ML analyses was evaluated using bootstrapping (BS) method for 1000 replicates. Bayesian inference (BI) was performed using a Markov Chain Monte Carlo (MCMC) algorithm to construct the topology of the tree [31]. Two MCMC chains were run from random trees for 10,000,000 generations and stopped when the average standard deviation of the split frequencies fell below 0.01. From each 1000 generations, the trees were saved. The first 25 % of trees were discarded as the burn-in phase of each analysis, and the posterior probabilities (BPP) were calculated to assess the remaining trees [32]. Phylograms were presented using Figtree v. 1.3.1 and processed by Adobe Illustrator CS v.5. Reference sequences were selected based on the type materials available in GenBank and published papers. The sequence data of the present study were deposited in GenBank, and the GenBank accession numbers of all accessions included in the phylogenetic analyses are listed in Table 3. 

## 3. Results

### 3.1. Results of PCR, Sequencing Using Different Primers

Both directed and nested PCRs were adopted for 24 DNA samples. The results of various combinations of primer pairs are presented in Table 4. 

Throughout the directed PCR procedure, using published primer pair ITS5/ITS4, only sample 1 was successfully sequenced, but it proved to be *Aspergillus* sp. For ITS1/ITS4, the sequencing result for sample 1 was the same as when using primer pair ITS5/ITS4, and sample 4 was *Gymnopus* sp. When using the ITS1/ITS2, samples 1 and 22 were all *Aspergillus* sp. The ITS3/ITS4 results were the same as using the primer pair ITS1/ITS4. 

When using specific primer pairs designed in this study for *G*. *sichuanense* (ITS1/ITSGs1-2, ITSGs1-1/ITS2, ITS3/ITSGs2-2, and ITSGs2-1/ITS4) from 24 DNA samples, we obtained 2, 6, 3, and 1 sequences respectively, 13 short fragments in total, which can assemble into one complete *G*. *sichuanense* sequences (HMAS 42798-d, Gene Bank number OP805623).

In the nested PCR experiment, ITS5/ITS4 was selected as the external primer pair, when using ITS1/ITS4 as the internal primer pair, the PCR amplification resulted in the presence of polymorphic bands. Only eight samples produced sequencing results (Tsingke Biological Technology). The results of the taxonomic groups belong to *Aspergillus*, *Astraeus*, *Cercospora*, *Cladosporium*, *Cryptococcus*, and *Pleosporales*. When using the ITS1/ITSGs4-4 primer pair as the internal primers, the agarose electrophoresis results were the same. 

**Figure 2:**
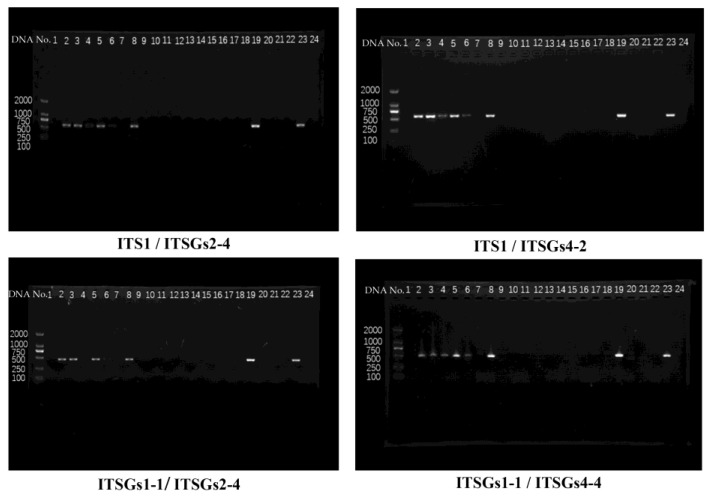
Gel electrophoresis image.

As for ITS1/ITSGs2-4, ITS1/ITSGs4-2, ITSGs1-1/ITSGs2-4, and ITSGs1-1/ITSGs4-4, the four electrophoretograms appeared to be consistent, each primer combination appeared as eight clear single target bands from the eight same samples (Figure 2). We selected ITS1/ITSGs4-2 (product size: 576 bp) and ITSGs1-1/ITSGs4-4 (product: size 488 bp) to perform the sequencing (Tsing Ke Biological Technology). We obtained 8 complete *G*. *sichuanense* sequences from each primer combination, 16 sequences in total were successfully sequenced.

### 3.2. Results of Genome Sequencing and Assembly

Genome sequencing produced 7,230,782 raw reads (1,084,617,300 bp) for HMAS 25103, resulting in 4,121,318 clean reads (612,283,701 bp) following filtration. Filtration refers to removing adapter sequences, low-quality reads, and reads higher than a certain proportion (10%) of N (ambiguous sites). Cleaned reads were assembled using MegaHit [33]. The assemblies contained 15,034 contigs (N50 = 6656 bp), the lengths of which were 31,314,762. The ITS sequence alignments included 61 species of *Ganoderma* were used as queries to search the possible target sequences obtained from the genome assemblies. A sole 556 bp nuclear ribosomal DNA (nrDNA) fragment was obtained, which contained a partial 5.8S ribosomal RNA gene, complete ITS2, and partial large subunit ribosomal RNA gene sequence. Additionally, the sequence was submitted to GenBank (Gene Bank number OP805628).

### 3.3. Results of Phylogenetic Analyses

**Figure 3:**
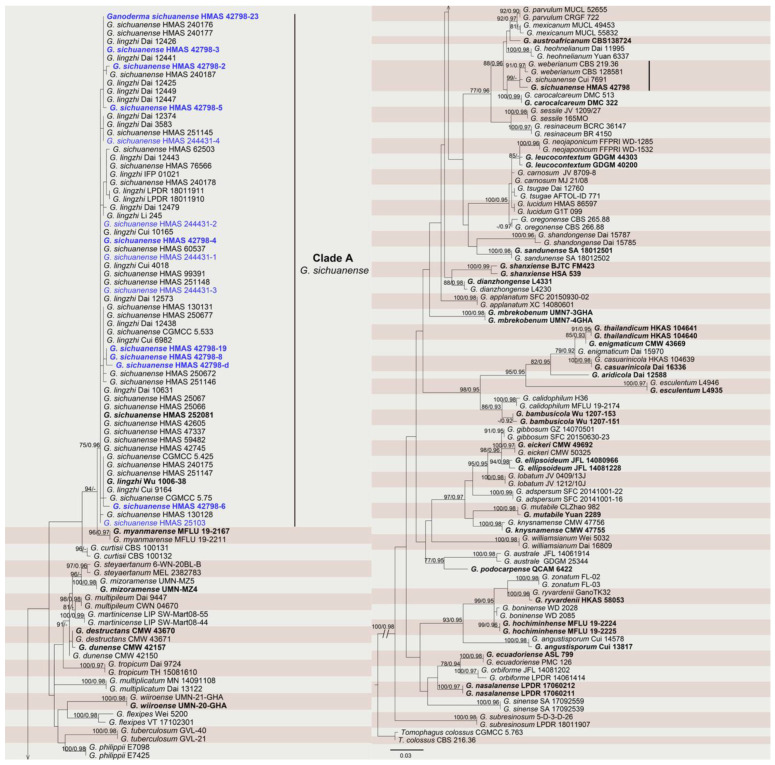
Phylogram of *Ganoderma* resulting from a maximum likelihood analysis based on the ITS gene. Numbers above the branches indicate ML bootstrap values (left, ML BS ≥ 75%) and Bayesian Posterior Probabilities (right, BPP ≥ 0.90). The tree is rooted with *Tomophagus colossus*. Sequences from the present study are marked in blue; type materials are bold.

The ITS dataset from 185 strains was analyzed to infer the interspecific relationships within *Ganoderma*. The sequences were clustered in 62 groups representing 61 known species of *Ganoderma* with 49 type isolates and 1 outgroup taxon *Tomophagus colossus*. The topologies resulting from the ML and BI analyses of the concatenated dataset were congruent. A total of 14 sequences obtained from the present study were formed in 1 individual clade (clade A) representing the species *G*. *sichuanese* (Figure 3).

### 3.4. Taxonomy

***Ganoderma sichuanense*** J.D. Zhao & X.Q. Zhang, Acta Mycol Sin. 2(3): 159 (1983)

Syn. *Ganoderma lingzhi* S.H. Wu, Y. Cao & Y.C. Dai, Fungal Divers. 56 (1): 54 (2012)

Materials examined—CHINA. Sichuan Province, Dukou (Panzhihua) City, Panzhihua Steel Plant, on the rotten wood of a broad-leaved tree, 1976, C.M. Li, 116 (Holotype HMAS 42798); CHINA. Panzhihua City, Renhe District, Zhongba Township, Xuefang Village, 26°25′11.43″ N, 101°40′17.54″ E, alt. 1458 m, purchased from a villager who gathered the specimens from mountainous areas surrounding the village, 14 October 2012, Y.J. Yao, B. Wang & X.C. Wang, 019 (HMAS 244431); CHINA. Beijing City, cultivated by Zhuang Deng, June 1959 (HMAS 25103).

Notes: A total of 43 sequences of *G*. *sichuanense* were used in this study and diverged into two different evolutionary branches. Except for two sequences (JQ781877 and JQ781878) grouped with *G*. *weberianum*, other sequences, including nine sequences obtained from the holotype of *G*. *sichuanense* (HMMAS 42798) in this study, were all gathered in clade A. Additionally, all sequences of the name “*G*. *lingzhi*” were also mixed in clade A. It is worth noting that clade A included many sequences from the type or pivotal materials of the two species mentioned above, as follows: (1) the sequence of *G*. *lingzhi* (Wu 1006-38) holotype and all other *G*. *lingzhi* sequences used in the paper of Cao et al. [20]; (2) nine sequences of the *G*. *sichuanense* (HMAS 42798) holotype obtained using two different independent PCR methods; (3) the sequence obtained from *G*. *sichuanense* (HMAS 252081) epitype [21] and four sequences obtained from different fruit bodies of topotype (HMAS 244431); (4) the sequence of first cultivated Lingzhi fruit bodies (HMAS 25103) in China. Our research suggests that clade A should be the authentic *G. sichuanense*. The new species *G*. *lingzhi* was proposed on the premise that ITS sequence JQ781877 was unquestionably obtained from the holotype of *G*. *sichuanense* [20], but the sequence JQ781877 was obtained only once [20,34]. The reasons why we insisted that the sequences of the holotype presented in this research are reliable are (1) the DNA samples of the *G*. *sichuanense* holotype were extracted using different methods, the CTAB method, the Wizard^®^ Genomic DNA Purification Kit (Promega, U.S.A.), and Chelex 100 Resin (Solarbio, China); (2) by directed and nested PCRs, the sequences obtained from different DNA samples from the *G*. *sichuanense* (HMAS 42798) holotype were clustered together in the phylogenetic analysis; (3) the first cultivated Lingzhi (HMAS 25103), which represents the widely cultivated Chinese *Ganoderma* species, was also clustered in the evolutionary branch of *G*. *sichuanense*. 

Complying with the International Code of Nomenclature for algae, fungi, and plants (Art.11.3), the earliest legitimate name of a taxon should be given priority [35]. *Ganoderma sichuanense* [19] has more preference than *G*. *lingzhi* [20]. The current results treat *G*. *lingzhi* as a later synonym of *G*. *sichuanense*.

## 4. Discussion

Although gene conversion [36] and unequal cross-over [37] are two of the most commonly proposed concerted evolution events [38], numerous studies have revealed that ITS is a multicopy gene and does not subscribe to evolution perfectly. Intra-strain and intra-species variations exist in several fungal taxa, such as *Fusarium* [39], *Laetiporus* [40], *Ophiocordyceps* [41], *Scutellospora* [42], *Xanthophyllomyces* [43], and also *Ganoderma* [44]. The results of our phylogenetic analysis demonstrated the ITS sequence heterogeneity within the holotype of *G*. *sichuanense* (HMAS 42798). This is the first report of ITS heterogeneity for *G*. *sichuanense*; heterogeneity occurs in three parts (ITS1, ITS2, and 5.8S) of the ITS region. This might explain why small divisions exist in the whole, large clade of *G*. *sichuanense* (Clade A). For nested PCR, the results are identical when using different primer pairs as second-round primer sets (ITS1/ITSGs4-2 and ITSGs1-1/ITSGs4-4). Therefore, we speculate that this phenomenon might be the nature of the species *G*. *sichuanense*.

**Figure 4:**
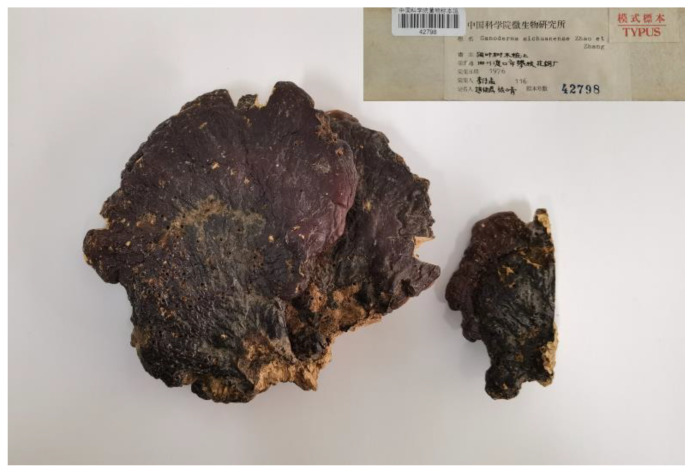
The holotype of *Ganoderma sichuanense* (HMAS 42798).

In the specimen box of the holotype, two fruit bodies existed (Figure 4). We can confirm that the sequence OP805618 (sample 5, HMAS 42798-5) was obtained from the big fruit body, and the sequence OP805619 (sample 6, HMAS 42798-6) was obtained from the small one. For the other DNA samples from the holotype, depending on the information on the lid of the DNA sample container and the lab notebook of the operator, we were unable to verify which fruit body was taken for (the DNA samples were extracted in 2010), but samples 5 and 6 ensured that all the fruit bodies of the holotype obtained ITS sequence successfully. 

We provided ITS sequences to perform a phylogenetic study based on the root of the long-standing discussion and the obtainable experimental results. The aim of the research was to resolve the problem of the scientific binomial for the widely cultivated Lingzhi in China, the discussion of which has centered around the controversial ITS sequence JQ781877 since 2012 [20]. We designed seven primers, using different PCR methods to successfully obtain repeatable and reliable ITS sequences from the holotype. Additionally, in our experiment, the attempts to amplify the other gene sequences from the holotype failed due to the largely disintegrated DNA. In some research papers concerning *Ganoderma*, even though other gene loci were applied, for the specimen HMAS 42798, only ITS sequence JQ781877 was available [20,22,45]. Therefore, based on the fact that the crux of the dispute was the key ITS sequence, and no other gene sequences of the holotype were available, the ITS phylogenic tree presented in this study can break the present deadlock. Though the ITS gene can perform species division in the genus *Ganoderma* [20,21] and is the most abundant gene region in *Ganodermataceae* [45], depending on a single gene does not address all the problems in the classification of *Ganoderma*. For the complex groups in *Ganoderma* or the higher rank classification of *Ganodermataceae*, multigene phylogenetic analyses are essential [22,45,46,47,48,49,50,51,52].

For the old specimens and DNA samples used in this research, any preserved DNA was present only in small amounts and in various states of degradation. Therefore, we explored various approaches for DNA amplification from the important, old samples, including designing new primers for directed and nested PCRs and genome sequencing. All the methods mentioned in this paper may also be applied to type material of other important species.

## Figures and Tables

**Table 1:** Primers designed in this study.

Name	Sequence 5′-3′	Tm (°C)	GC%	Length	Location
ITSGs1-1	TAC TGT GGG CTT CAG ATT GC	56.0	55.0	20	ITS1
ITSGs1-2	GTG CCT CGC AAT CTG AAG C	58.9	57.9	19	ITS1
ITSGs2-1	TTA TCG GTC GGC TCC TCT TA	57.7	50.0	20	ITS2
ITSGs2-2	AAG AGG AGC CGA CCG ATA AC	58.4	55.0	20	ITS2
ITSGs2-4	AGC TGT CTT ATA AGA CGG T	51.6	36.4	22	ITS2
ITSGs4-2	CAG GTC ATA AAG CTG TCT TAT	48.4	38.1	21	ITS2 & 28s
ITSGs4-4	GTC CTA CCT GAT TTG AGG TCA	54	47.6	21	28s

**Table 2:** Nested PCR primer combinations.

Primers for the First Round PCR	Primers for the Second Round PCR
ITS5/ITS4	ITS1/ITS4
	ITS1/ITSGs2-4
	ITS1/ITSGs4-2
	ITS1/ITSGs4-4
	ITSGs1-1/ITSGs2-4
	ITSGs1-1/ITSGs4-4

**Table 3:** Strains and GenBank accession numbers used in this study.

Species	Voucher/Strain	Origin	Accession Number (ITS)
*Ganoderma adspersum* (Schulzer) Donk	SFC 20141001-16	Korea	KY364251
*G. adspersum*	SFC 20141001-22	Korea	KY364252
*G. angustisporum* J.H. Xing, B.K. Cui & Y.C. Dai	Cui 13817 (holotype)	China	MG279170
*G. angustisporum*	Cui 14578	China	MG279171
*G. applanatum* (Pers.) Pat.	SFC 20150930-02	Korea	KY364258
*G. applanatum*	XC 14080601	China	MK345426
*G. aridicola* J.H. Xing & B.K. Cui	Dai 12588 (holotype)	South Africa	KU572491
*G. australe* (Fr.) Pat.	GDGM 25344	China	JX195198
*G. australe*	GACP 14061914	China	MK345428
*G. austroafricanum* M.P.A. Coetzee, M.J. Wingf., Marinc. & Blanchette	CBS 138724 (ex-type)	South Africa	KM507324
*G. bambusicola* Sheng H. Wu, C.L. Chern & T. Hatt.	Wu 1207-151 (holotype)	China	MN957781
*G. bambusicola*	Wu 1207-153 (paratype)	China	MN957783
*G. boninense* Pat.	WD 2028	Japan	KJ143905
*G. boninense*	WD 2085	Japan	KJ143906
*G. calidophilum* J.D. Zhao, L.W. Hsu & X.Q. Zhang	MFLU 19-2174	China	MN398337
*G. calidophilum*	H36	China	MW750241
*G. carnosum* Pat.	JV 8709/8	Czech R	KU572493
*G. carnosum*	MJ 21/08	Czech R	KU572492
*G. carocalcareum* Douanla-Meli	DMC 322 (holotype)	Cameroon	EU089969
*G. carocalcareum*	DMC 513	Cameroon	EU089970
*G. casuarinicola* J.H. Xing, B.K. Cui & Y.C. Dai	Dai 16336 (holotype)	China	MG279173
*G. casuarinicola*	HKAS 104639	Thailand	MK817650
*G. curtisii* (Berk.) Murrill	CBS 100131	USA	JQ781848
*G. curtisii*	CBS 100132	USA	JQ781849
*G. destructans* M.P.A. Coetzee, Marinc. & M.J.Wingf.	CMW 43670 (ex-type)	South Africa	KR183856
*G. destructans*	CMW 43671	South Africa	KR183857
*G. dianzhongense* J. He, H.Y. Su & S.H. Li	L4331 (holotype)	China	MW750237
*G. dianzhongense*	L4230	China	MW750236
*G. dunense* Tchotet, Rajchenb. & Jol. Roux	CMW 42157 (holotype)	South Africa	MG020255
*G. dunense*	CMW 42150	South Africa	MG020249
*G. ecuadoriense* A. Salazar, C.W. Barnes & Ordoñez	ASL 799 (holotype)	Ecuador	KU128524
*G. ecuadoriense*	PMC 126	Ecuador	KU128525
*G. eickeri* Tchotet, M.P.A. Coetzee, Rajchenb. & Jol. Roux	CMW 49692 (holotype)	South Africa	MH571690
*G. eickeri*	CMW 50325	South Africa	MH571689
*G. ellipsoideum* Hapuar., T.C. Wen & K.D. Hyde	GACP 1408966 (holotype)	China	MH106867
*G. ellipsoideum*	GACP 14081215 (paratype)	China	MH106886
*G. enigmaticum* M.P.A. Coetzee, Marinc. & M.J.Wingf.	CMW 43669 (ex-type)	South Africa	KR183855
*G. enigmaticum*	Dai 15970	South Africa	KU572486
* G. esculentum * J. He & S.H. Li	L4935 (holotype)	China	MW750242
* G. esculentum *	L4946	China	MW750243
*G. flexipes* Pat.	VT 17102301	Vietnam	MK345430
*G. flexipes*	Wei 5200	China	JN383978
*G. gibbosum* (Cooke) Pat.	SFC 20150630-23	Korea	KY364264
*G. gibbosum*	GZ 14070501	China	MK345432
*G. heohnelianum* Bres.	Yuan 6337	China	MG279160
*G. heohnelianum*	Dai 11995	China	KU219988
* G. hochiminhense * Karunarathna, Mortimer, Huyen & Luangharn	MFLU 19-2224 (holotype)	Vietnam	MN398324
*G. hochiminhense*	MFLU 19-2225 (paratype)	Vietnam	MN396662
* G. knysnamense * Tchotet, M.P.A. Coetzee, Rajchenb. & Jol. Roux	CMW 47755 (ex-type)	South Africa	MH571681
*G. knysnamense*	CMW 47756	South Africa	MH571684
*G. leucocontextum* T.H. Li, W.Q. Deng, Sheng H. Wu, Dong M. Wang & H.P. Hu	GDGM 40200 (holotype)	China	KF011548
*G. leucocontextum*	GDGM 44303(paratype)	China	KJ027607
*G*. *lingzhi* Sheng H. Wu, Y. Cao & Y.C. Dai	Wu 1006-38 (holotype)	China	JQ781858
*G*. *lingzhi*	Cui 4018	China	JQ781856
*G*. *lingzhi*	Cui 10165	China	JQ781857
*G*. *lingzhi*	Cui 9164	China	JQ781859
*G*. *lingzhi*	Dai 10631	China	JQ781860
*G. lingzhi*	Dai 12438	China	JQ781861
*G*. *lingzhi*	Dai 12479	China	JQ781864
*G*. *lingzhi*	IFP 01021	China	JQ781865
*G*. *lingzhi*	Dai 12443	China	JQ781866
*G*. *lingzhi*	Dai 12374	China	JQ781867
*G*. *lingzhi*	Dai 3583	China	JQ781868
*G*. *lingzhi*	Dai 12441	China	JQ781869
*G*. *lingzhi*	Dai 12426	China	JQ781870
*G*. *lingzhi*	Dai 12425	China	JQ781871
*G*. *lingzhi*	Dai 12447	China	JQ781872
*G*. *lingzhi*	Dai 12449	China	JQ781873
*G*. *lingzhi*	Cui 6982	China	JQ781862
*G*. *lingzhi*	Dai 12573	China	JQ781855
*G*. *lingzhi*	Li245	China	JQ781863
*G*. *lingzhi*	LPDR 18011910	Laos	MK345437
*G*. *lingzhi*	LPDR 18011911	Laos	MK345438
*G. lobatum* (Cooke) G.F. Atk.	JV1212/10J	USA	KF605676
*G. lobatum*	JV0409/13J	USA	KF605675
*G. lucidum* (Curtis) P. Karst.	HMAS 86597	UK	AY884176
*G. lucidum*	G1T 099	Italy	AM269773
*G. martinicense* Welti & Courtec	LIP SW-Mart08-44	France	KF963257
*G. martinicense*	LIP SW-Mart08-55	France	KF963256
*G. mbrekobenum* E.C. Otto, Blanchette, Held, C.W. Barnes & Obodai	UMN7-3 GHA (holotype)	Ghana	KX000896
*G. mbrekobenum*	UMN7-4 GHA (paratype)	Ghana	KX000898
*G. mexicanum Pat.*	MUCL 49453	Martinique	MK531811
*G. mexicanum*	MUCL 55832	Martinique	MK531815
*G. mizoramense* Zothanz., Blanchette, Held & C.W. Barnes	UMN-MZ4 (holotype)	India	KY643750
*G. mizoramense*	UMN-MZ5	India	KY643751
*G. multipileum* Ding Hou	CWN 04670	China	KJ143913
*G. multipileum*	Dai 9447	China	KJ143914
*G. multiplicatum* (Mont.) Pat.	Dai 13122	China	KU572488
*G. multiplicatum*	MN 14091108	Myanmar	MK345440
*G. mutabile* Y. Cao & H.S. Yuan	Yuan 2289 (holotype)	China	JN383977
*G. mutabile*	CLZhao 982	China	MG231527
*G. myanmarense* Karunarathna, Mortimer & Luangharn	MFLU 19-2167 ((holotype)	Myanmar	MN396330
*G. myanmarense*	MFLU 19-2211 (paratype)	Myanmar	MN396329
*G. nasalanense* Hapuar., Pheng., & K.D. Hyde.	LPDR 17060211 (holotype)	Laos	MK345441
*G. nasalanense*	LPDR 17060212 (paratype)	Laos	MK345442
*G. neojaponicum* Imazeki	FFPRI WD-1285	Japan	MN957784
*G. neojaponicum*	FFPRI WD-1532	Japan	MN957785
*G. orbiforme* (Fr.) Ryvarden	JFL 14081202	China	MK345445
*G. orbiforme*	GACP 14061414	Laos	MK345446
*G. oregonense* Murrill	CBS 265.88	USA	JQ781875
*G. oregonense*	CBS 266.88	USA	JQ781876
*G. parvulum* Murrill	MUCL 47096	Cuba	MK554783
*G. parvulum*	MUCL 52655	French Guiana	MK554770
*G. philippii* (Bres. & Henn. ex Sacc.) Bres.	E7098	Malaysia	AJ536662
*G. philippii*	E7425	Malaysia	AJ608713
*G. podocarpense* J.A. Flores, C.W. Barnes & Ordoñez	QCAM 6422 (holotype)	Ecuador	MF796661
*G. resinaceum* Boud.	BCRC 36147	Netherlands	KJ143916
*G. resinaceum*	BR 4150	France	KJ143915
*G. ryvardenii* Tonjock & Mih	HKAS 58053 (holotype)	Cameroon	HM138671
*G. ryvardenii*	GanoTK32	Cameroon	JN105698
*G. sandunense* Hapuar., T.C. Wen & K.D. Hyde.	SA 18012501 (holotype)	China	MK345450
*G. sandunense*	SA 18012502	China	MK345451
*G. sessile* Murrill	JV 1209/27	USA	KF605630
*G. sessile*	165MO	USA	MG654312
*G. shandongense* J.D. Zhao & L.W. Xu	Dai 15785	China	MG279190
*G. shandongense*	Dai 15787	China	MG279191
*G. shanxiense* L. Fan & H. Liu	BJTC FM423 (holotype)	China	MK764268
*G. shanxiense*	HSA 539 (paratype)	China	MK764269
***G. sichuanense* J.D. Zhao & X.Q. Zhang**	**HMAS 42798-2 (holotype)**	**China**	**OP805615**
***G. sichuanense***	**HMAS 42798-3 (holotype)**	**China**	**OP805616**
***G. sichuanense***	**HMAS 42798-4 (holotype)**	**China**	**OP805617**
***G. sichuanense***	**HMAS 42798-5 (holotype)**	**China**	**OP805618**
***G. sichuanense***	**HMAS 42798-6 (holotype)**	**China**	**OP805619**
***G. sichuanense***	**HMAS 42798-8 (holotype)**	**China**	**OP805620**
***G. sichuanense***	**HMAS 42798-19 (holotype)**	**China**	**OP805621**
***G. sichuanense***	**HMAS 42798-23 (holotype)**	**China**	**OP805622**
***G. sichuanense***	**HMAS 42798-d (holotype)**	**China**	**OP805623**
***G. sichuanense***	**HMAS 244431-1**	**China**	**OP805624**
***G. sichuanense***	**HMAS 244431-2**	**China**	**OP805625**
***G. sichuanense***	**HMAS 244431-3**	**China**	**OP805626**
***G. sichuanense***	**HMAS 244431-4**	**China**	**OP805627**
***G. sichuanense***	**HMAS 25103**	**China**	**OP805628**
*G. sichuanense*	HMAS 252081 (epitype)	China	KC662402
*G. sichuanense*	HMAS 25066	China	JN197275
*G. sichuanense*	HMAS 25067	China	JN197276
*G. sichuanense*	HMAS 42605	China	JN197277
*G. sichuanense*	HMAS 42745	China	JN197278
*G. sichuanense*	HMAS 47337	China	JN197279
*G. sichuanense*	HMAS 59482	China	JN197280
*G. sichuanense*	HMAS 60537	China	JN197281
*G. sichuanense*	HMAS 62503	China	JF915405
*G. sichuanense*	HMAS 76566	China	JF915406
*G. sichuanense*	HMAS 99391	China	JF915407
*G. sichuanense*	HMAS 130131	China	JF915408
*G. sichuanense*	HMAS 240175	China	JF915393
*G. sichuanense*	HMAS 240176	China	JF915394
*G. sichuanense*	HMAS 240177	China	JF915395
*G. sichuanense*	HMAS 240178	China	JF915396
*G. sichuanense*	HMAS 240187	China	JF915397
*G. sichuanense*	HMAS 250672	China	JF915398
*G. sichuanense*	HMAS 250677	China	JF915399
*G. sichuanense*	HMAS 251145	China	JF915400
*G. sichuanense*	HMAS 251146	China	JF915401
*G. sichuanense*	HMAS 251147	China	JF915402
*G. sichuanense*	HMAS 251148	China	JF915403
*G. sichuanense*	HMAS130128	China	JF915404
*G. sichuanense*	CGMCC 5.75	China	JN197282
*G. sichuanense*	CGMCC 5.425	China	JN197283
*G. sichuanense*	CGMCC 5.533	China	JN197284
*G. sichuanense*	Cui 7691	China	JQ781878
*G. sichuanense*	HMAS 42798 (holotype)	China	JQ781877
*G. sinense* J.D. Zhao, L.W. Hsu & X.Q. Zhang	SA 17092559	China	MK345452
*G. sinense*	SA 17092539	China	MK345453
*G. steyaertanum* B.J. Sm. & Sivasith.	MEL:2382783	Australia	KP012964
*G. steyaertanum*	6-WN-20BL-B	Indonesia	KJ654462
*G. subresinosum* (Murrill) C.J. Humphrey	5-D-3-D-26	Indonesia	KJ654467
*G. subresinosum*	LPDR 18011907	Laos	MK345455
*G. thailandicum* Luangharn, P.E. Mortimer, Karun. & J.C. Xu	HKAS 104640 (holotype)	Thailand	MK848681
*G. thailandicum*	HKAS 104641 (paratype)	Thailand	MK848682
*G. tropicum* (Jungh.) Bres.	Dai 9724	China	JQ781879
*G. tropicum*	TH 15081610	Thailand	MK345456
*G. tsugae* Murrill	Dai 12760	USA	KJ143920
*G. tsugae*	AFTOL-ID 771	--	DQ206985
*G. tuberculosum* Murrill	GVL-21	Mexico	MT232639
*G. tuberculosum*	GVL-40	Mexico	MT232634
*G. weberianum* (Bres. & Henn. ex Sacc.) Steyaert	CBS 219.36	Philippines	JQ520219
*G. weberianum*	CBS 128581	Taiwan, China	MK603805
*G. wiiroense* E.C. Otto, Blanchette, C.W. Barnes &Held	UMN-20-GHA (paratype)	Ghana	KT952361
*G. wiiroense*	UMN-21-GHA	Ghana	KT952363
*G. williamsianum* Murrill	Dai 16809	China	MG279183
*G. williamsianum*	Wei 5032	China	KU219994
*G. zonatum* Murrill	FL-02	USA	KJ143921
*G. zonatum*	FL-03	USA	KJ143922
*Tomophagus colossus* (Fr.) Murrill	CBS 216.36	Philippines	Z37071&Z37091
*T.s colossus*	CGMCC 5.763	Philippines	JQ081068

Note: --, not applicable; sequences obtained in the present study are in black and bold.

**Table 4:** The results of PCR amplification.

Method	Primer Pair	Results	Product Size (bp)
Directed PCR	ITS5/ITS4	*	654
	ITS1/ITS4	*	633
	ITS1/ITS2	*	266
	ITS3/ITS4	*	367
	ITS1/ITSGs1-2	+	116
	ITSGs1-1/ITS2	+	157
	ITS3/ITSGs2-2	+	180
	ITSGs2-1/ITS4	+	176
Nested PCR	First round PCR		
	ITS5/ITS4	*	654
	Second round PCR		
	ITS1/ITS4	*	633
	ITS1/ITSGs4-4	*	597
	ITS1/ITSGs2-4	+	567
	ITS1/ITSGs4-2	+	576
	ITSGs1-1/ITSGs2-4	+	458
	ITSGs1-1/ITSGs4-4	+	488

Note: + specific PCR product; * nonspecific PCR product.

## Data Availability

The sequences in the present study were submitted to the NCBI website (https://www.ncbi.nlm.nih.gov/), and the accession numbers are listed in Table 3.
